# 1858. Examining the Racial Differences in Polymicrobial Bacteremia Infections in an Area of Varying Social Vulnerability

**DOI:** 10.1093/ofid/ofad500.1686

**Published:** 2023-11-27

**Authors:** Karen K Tan, Jacinda C Abdul-Mutakabbir

**Affiliations:** Loma Linda University Medical Center, Loma Linda, California; University of California San Diego, CA

## Abstract

**Background:**

Polymicrobial bacteremia (PMB) is associated with heightened disease severity and an increased burden of comorbidities has been identified as a key risk factor. Of interest, racially and ethnically minoritized (REM) individuals that reside in areas of high vulnerability have been shown to be more likely than their non-racially and ethnically minoritized (n-REM) counterparts to be diagnosed with chronic comorbidities. Nonetheless, there is a cessation of data that examines the confluence of these factors on PMB infection outcomes. This study aims to describe the racial differences in the outcomes of patients diagnosed with PMB in an area of varying social vulnerability.

**Methods:**

This is an IRB-approved, retrospective cohort study of adult patients hospitalized at Loma Linda University Medical Center between January 2018 and April 2021. Positive polymicrobial blood culture samples were identified with the use of Microscan Bacterial Identification Panel. Demographics (including race/ethnicity), comorbid conditions, sepsis incidence, and infection source were collected through chart review. The Centers for Disease Control and Prevention (CDC) SVI scoring tool was used to determine the social vulnerability index (SVI). A P value of ≤ 0.05 was defined as statistically significant.

**Results:**

A total of 70 patients REM (60%, 42/70) and non-REM (40%, 28/70) among PMB gram-negative (GN) and gram-positive (GP) cultures collected from hospitalized adult patients were included. Hispanic/Latino was the most prominent REM group (59.5%) and overall, REM patients were more likely to be classified as “highly vulnerable” when compared to non-REM patients (47.6% vs 42.9%). Also, REM patients were younger (61.5 years vs. 65.1 years) and were more likely to have underlying comorbidities (diabetes mellitus (47.6% vs 21.4%; p=0.026 and moderate to severe chronic kidney disease (28.8% vs 14.3%; p= 0.329). Additionally, REM patients were more likely to develop sepsis and severe sepsis (45.2% vs. 25.0%; p= 0.13 and 40.6% vs 35.7%, p=0.804), respectively, though there was no difference in mortality.

**Conclusion:**

Ultimately, further research is needed to understand the confluence of race/ethnicity and social vulnerability factors on PMB clinical outcomes.

**Disclosures:**

**Jacinda C. Abdul-Mutakabbir, PharmD, MPH**, Entasis: Advisor/Consultant|Entasis: Honoraria|Shionogi: Advisor/Consultant|Shionogi: Honoraria

